# Bioimpedance-Based Measurements of In Vitro Biological Cell Barrier Integrity: A Review and Framework for the Acquisition and Analysis Strategies

**DOI:** 10.3390/s26082477

**Published:** 2026-04-17

**Authors:** Shaginth Sivakumar, João Pinheiro Marques, Adrien Roux

**Affiliations:** School of Engineering, Architecture and Landscape of Geneva (HEPIA), HES-SO—University of Applied Sciences and Arts Western Switzerland, Campus Biotech, 9 Chemin des Mines, CH-1202 Geneva, Switzerland; shaginth.sivakumar@hesge.ch (S.S.); joao.marques@hesge.ch (J.P.M.)

**Keywords:** *in vitro* cell barrier, trans-epithelial/endothelial electrical resistance (*TEER*), electrical impedance spectroscopy (EIS), electrode–electrolyte interface, new approach methodologies (NAMs), bioimpedance

## Abstract

**Highlights:**

This review targets two complementary audiences in in vitro cell barrier research. For biologists and experimental researchers, it clarifies impedance-derived cell barrier metrics and provides practical guidance for applying a three-level analytical hierarchy adapted to experimental objectives. For engineers and device developers, it covers the emerging needs in electrode design, frequency coverage, and measurement strategies for reliable impedance acquisition. The review further emphasizes methodological standardization, analytical transparency, and harmonized reporting to improve measurement reliability, reproducibility, and cross-platform comparability within New Approach Methodologies (NAMs)-oriented research frameworks.

**What are the main findings?**

- Single-frequency Trans-Epithelial/Endothelial Electrical Resistance (*TEER*) reflects the combined response of a coupled electrochemical system and impedance magnitude measurements do not only represent tight junction-mediated resistance as they neglect capacitive contributions of the other components of the system.
- Phase-resolved electrical impedance spectroscopy enables separation of resistive and capacitive contributions and supports a structured, three-level analytical strategy for cell barrier monitoring.

**What are the implications of the main findings?**

- Monitoring of TEER is increasingly used as a complementary readout within NAMs in the field of drug delivery, toxicity, and pharmacokinetics.
- Researchers should report all parameters of their measuring device, acquisition setup, and analysis methodology to improve reproducibility, inter-platform comparability, and integration of cell barrier models within NAM-based research workflows.
- Single-frequency reported *TEER* approximates tight junctions’ resistance influenced by capacitive contributions of the acquisition setup and cell culture conditions. Using Electrical Impedance Spectroscopy (EIS), researchers can isolate these variables and perform long-term experimentation.

**Abstract:**

In vitro cell barrier models have been increasingly integrated into pharmaceutical and academic research pipelines to evaluate drug safety and drug delivery due to a shift towards New Approach Methodologies (NAMs) in research and regulatory safety assessment. Such models require reliable and interpretable functional readouts. Bioimpedance-based monitoring, particularly transepithelial/endothelial electrical resistance (*TEER*), is a widely adopted readout due to its non-invasive and real-time capabilities. However, substantial variability arises from differences in measurement settings, frequency selection, electrode configuration, impedance measuring techniques, and data analysis strategies. In numerous studies, *TEER* is approximated from single-frequency impedance magnitude measurements, which do not isolate the resistive component associated with tight junction-mediated paracellular transport but instead reflect the combined response of a coupled electrochemical system. This review clarifies impedance measuring techniques and systematically analyzes impedance-based measurement and analysis strategies for in vitro biological cell barrier integrity. We compare mono-frequency and broadband acquisition approaches, examine the influence of electrode–electrolyte interfaces, electrode geometry, and culture configuration, and evaluate equivalent circuit modeling and phase-resolved electrical impedance spectroscopy (EIS). Based on this comparison, we propose a three-level analytical hierarchy adapted to experimental objectives and instrumentation constraints. We conclude that phase-informed impedance analysis and harmonized reporting are essential to improve measurement reproducibility, inter-platform comparability, and integration of impedance-derived cell barrier assessment within NAMs-oriented research workflows.

## 1. Introduction

New Approach Methodologies (NAMs) are human-relevant methods used to assess biological effects without relying on animal models. These methods, which include *in vitro* systems, *in silico* models, and other approaches, are increasingly transforming the pharmaceutical and academic research landscape in response to the limitations of traditional animal models [1,2] in fields such as preclinical drug development, drug safety, and toxicity assessment. Species-specific differences in organ configuration, vascularization, and endothelial and epithelial cell barrier properties, which often alter drug delivery, lead to inconsistent pharmacokinetics and therapeutic outcomes in humans [3,4]. In addition to translational failure, animal experimental costs and regulatory evolution under the principles of replacement, reduction, and refinement (3Rs) of animal experimentation are accelerating the development of NAMs, particularly for drug toxicity and safety assessment [5,6].

Increasing physiologically relevant *in vitro* cell barrier systems with reliable functional readouts are progressively required for drug development and toxicology [1,7]. In this context, epithelial and endothelial barrier models, including human-induced pluripotent stem-cell-derived systems, are developed to recapitulate selective transport interfaces such as the blood–brain barrier, pulmonary air–blood interface, intestinal epithelium or renal filtration barrier, which regulate molecular transport, ionic exchange and drug permeability across tissues, and to support the development of increasingly complex advanced *in vitro* platforms that better reproduce organ-specific physiological functions [3,4,8].

To monitor epithelial and endothelial cell barrier integrity, Trans-Epithelial/Endothelial Electrical Resistance (*TEER*) measurement has become a widely adopted methodology [9,10,11]. Other methodologies include apparent permeability assays and specific protein localization assessment through immunolabeling. The popularity of *TEER* measurement over other methodologies derives from its non-invasive nature and compatibility with real-time monitoring across cell-culture inserts, well plates, and Organ-On-Chip (OOC) platforms [9,12,13]. However, persistent ambiguity remains regarding what *TEER* represents. From an electrical standpoint, *TEER* corresponds to the resistive component of the electrical impedance measured in a biological system, commonly referred to as bioimpedance, and is therefore derived from the frequency-dependent response of a coupled electrochemical system [10,14,15].

In biological practice, *TEER* is expressed as a surface-normalized resistance (Ω·cm^2^), obtained by multiplying the measured resistance by the culture area to reduce geometrical dependence. This convention implicitly assumes uniform current distribution and homogeneous barrier properties. These approximations may not strictly hold in complex, miniaturized, or microfluidic systems.

This single frequency measurement does not isolate the resistive contribution associated with paracellular transport through tight junctions, as the measured signal arises from a heterogeneous and dynamic electrochemical system in which the biological layer is coupled to electrode–electrolyte interfaces and background media [15,16]. Although surface normalization of impedance magnitude provides a practical reporting convention, it does not capture the full electrochemical complexity of the system. The impedance response integrates both resistive and capacitive contributions that evolve with cell shape, confluency, tight junction organization, medium composition, and electrode behavior [16,17,18], resulting in a configuration-dependent descriptor of barrier status rather than a direct quantification of junctional tightness [10,14,15].

To mitigate this limitation, electrical impedance spectroscopy (EIS) records the complex impedance response across a broadband frequency range (typically 1 Hz to 100 kHz) by applying a small-amplitude sinusoidal excitation while sweeping the frequency [14,16]. Using EIS, *TEER* is determined as a model-derived resistive parameter rather than approximated from a single impedance magnitude that neglects phase information and merges the resistive and capacitive contributions of the cell barrier [11,19].

Numerous studies have measured cell barrier integrity using different recording devices, frequency selections, electrode configurations, electrode materials, cultureware formats, and data analysis procedures, leading to substantial variability even for similar cell barrier models [10,20,21]. This variability contributes to the difficulty of comparing *TEER* values across laboratories and limits the reliability of impedance-derived readouts as standardized endpoints for toxicology and drug development workflows. In response, recent efforts have proposed minimum reporting information for *TEER* assays, reflecting a growing need for methodological harmonization in the NAMs context [22].

This review aims to clarify impedance-based epithelial and endothelial cell barrier monitoring and explicitly distinguishes biological cell barrier integrity from impedance-derived parameters. We report that *TEER* should be interpreted within complex impedance analysis rather than approximated from single-frequency magnitude readings. By systematically comparing measurement configurations and analytical strategies, including commercially available *TEER*/EIS platforms (Table 1) and electrode implementations used in cell barrier monitoring (Table 2), we propose a structured framework to analyze impedance data across acquisition methods and to improve reproducibility, comparability, and integration of impedance-based cell barrier assessment within NAMs-oriented research.

## 2. Physical Foundations of Impedance-Based Cell Barrier Monitoring

### 2.1. In Vitro Biological Cell Barriers as Electrochemical Systems

An *in vitro* epithelial or endothelial cell barrier is not only a biological construct but also a heterogeneous electrochemical system defined by ionic transport, dielectric polarization, and culture configuration [15,16,23]. The properties of the surrounding medium also influence the measured impedance. In particular, the ionic composition, conductivity, and physicochemical properties of the electrolyte govern ionic mobility and therefore the electrical response of the system. Variations in ion concentration, pH, protein content, or temperature can modify the measured impedance. Changes in viscosity may also affect ionic mobility, leading to increased resistivity and consequently higher impedance, especially in the bulk medium contribution represented by Z_Background_ in Equation (8). These measurements are performed in ionic cell culture media under controlled physiological conditions, which contribute to the electrochemical response of the system. These effects are independent of the biological barrier itself and may contribute to variability in impedance measurements over time or across experimental conditions.

When a confluent cell monolayer forms, specialized transmembrane proteins assemble into tight junctions that regulate selective permeability and control molecular exchange across the cell barrier [8]. These tight junction complexes are composed of multiple protein families, including claudins, occludin, junctional adhesion molecules (JAMs) and zonula occludens (ZOs) scaffolding proteins, which collectively regulate paracellular electrical conduction across the cell barrier.

**Figure 1:**
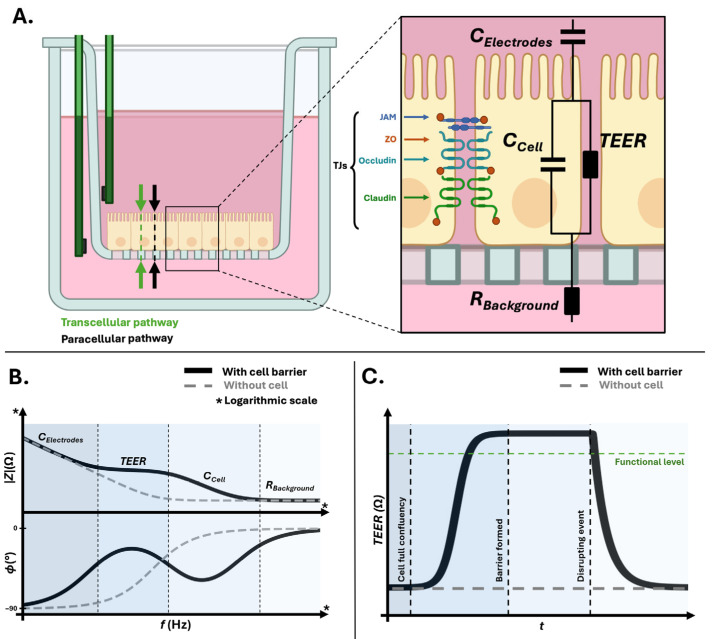
Electrical modeling and dynamic impedance behavior of an *in vitro* biological barrier. (**A**) Schematic representation of an *in vitro* cell barrier cultured on a porous membrane within a cell culture insert. Upon confluence, tight junction (TJ) complexes composed of claudins, occludins, junctional adhesion molecules (JAMs), and *zonula occludens* (ZOs) proteins establish barrier function. Bioimpedance is monitored using two electrodes that apply a small sinusoidal excitation across the cell barrier to assess its integrity through the paracellular pathway (black arrow) and the transcellular pathway (green arrow). The corresponding electrical equivalent model includes the electrode capacitance ($C_{Electrodes}$), the paracellular resistance associated to tight junction formation (TEER), the cell membrane capacitance representing the transcellular pathway ($C_{Cell}$), and the background resistance ($R_{Background}$). (**B**) Idealized Bode magnitude and phase plots illustrating the frequency-dependent contributions of the electrical equivalent circuit components. (**C**) Expected *TEER* over time after cell full confluency, barrier formation and disruption, highlighting the distinction between the background (without cell) level and the functional level of cell barrier *TEER*.

Cell barrier transport is primarily defined by two pathways: the paracellular pathway, which is modulated by tight junctions and the transcellular pathway, which involves transport across the cell membrane (Figure 1A) [3,4,8].

The paracellular pathway, controlled by tight junction organization, behaves predominantly as a resistive element (Figure 1A). The resistance R of a culture medium is defined as:

(1) $$R=\rho \frac{l}{A}$$

where ρ is the resistivity, l the effective current path length, and A the cross-sectional area. This fundamental relation highlights that resistance is intrinsically dependent on geometric parameters and current distribution. Implicitly, a homogeneous current density over the conductive cross-section is assumed (Equation (1)). In microfluidic systems or in configurations using non-planar or asymmetrically positioned electrodes, current density distribution is rarely uniform. Field lines become geometry-dependent, leading to spatially heterogeneous current pathways that deviate from the homogeneous assumption implicit in Equation (1) [18,23].

In contrast, the transcellular pathway is limited by the insulating properties of lipid bilayers and behaves predominantly as a capacitive element (Figure 1A). At first approximation and for alignment with electrode modeling, the cell membrane can be represented as a parallel-plate capacitor C:

(2) $$C=\varepsilon_{0}\varepsilon_{r}\frac{A}{d}$$

where d is the cell membrane thickness, $\varepsilon_{0}$ the absolute permittivity, and $\varepsilon_{r}$ the insulating layer relative permittivity. In a confluent monolayer, each cell presents apical and basolateral membranes. Electrically, these cell membranes are coupled in series at the single-cell level, while the ensemble of cells forms a distributed capacitive network at the tissue scale.

Cell barrier electrical behavior arises from the coupling between resistive junctional conduction and capacitive membrane polarization. Importantly, the cell layer is not electrically isolated as porous membranes, coatings, culture medium compartments, and electrode interfaces are electrically coupled to the monolayer (Figure 1A). The measured signal thus reflects the entire electrochemical system rather than the biological layer alone [14,19,23].

### 2.2. Frequency-Dependent Impedance Response

When a biological cell barrier is subjected to small alternating electrical excitations, the applied current I(ω) generates a voltage response U(ω). In impedance spectroscopy, their ratio defines the complex impedance:

(3) $$Z(\omega )=\frac{U(\omega )}{I(\omega )}=Z^{\prime }(\omega )+jZ^{\prime \prime }(\omega )$$

where $Z^{\prime }$ represents the resistive contribution associated with energy dissipation through ionic transport, $Z^{\prime \prime }$ represents the reactive contribution associated with energy storage mechanisms, and j denotes the imaginary unit defined by $j^{2}=-1$ [16]. For practical interpretation, impedance magnitude ∣Z∣ and phase ϕ are calculated as follows:

(4) $$|Z|=\sqrt{(Z^{\prime }{)}^{2}+(Z^{\prime \prime }{)}^{2}}$$

(5) $$\phi =arctan\left(\frac{Z^{\prime \prime }}{Z^{\prime }}\right)$$

For a cell barrier submitted to alternating current during impedance monitoring, the ideal electrochemical system is essentially based on a combination of resistive and capacitive elements. An ideal resistive element contributes only to the real component:

(6) $$Z_{R}(\omega )=R$$

whereas an ideal capacitive element contributes only to the imaginary component:

(7) $$Z_{C}(\omega )=\frac{1}{j\omega C}$$

This formula explicit that impedance is inherently frequency-dependent whenever capacitive contributions are present. As a result, any single-frequency measurement represents only a partial projection of the frequency-dependent electrochemical system (Equations (3)–(7)).

### 2.3. Minimal Equivalent Representation

Under simplified conditions, the impedance of a cell barrier measured between two electrodes positioned across a cell layer can be represented by the minimal equivalent structure:

(8) $$Z_{eq}=Z_{electrode}+(Z_{TEER}∥Z_{cell})+Z_{background}$$

where $Z_{TEER}$ characterizes paracellular conduction through tight junctions, $Z_{cell}$ represents the capacitive contribution of the cell layer, $Z_{electrode}$ characterizes the electrode–electrolyte interface, and $Z_{background}$ represents residual contributions from culture supports and the measurement configuration (Figure 1A).

For idealized elements:

(9) $$Z_{TEER}=TEER$$

(10) $$Z_{cell}=\frac{1}{j\omega C_{cell}}$$

The parallel combination is expressed as:

(11) $$Z_{TEER}∥Z_{cell}=\frac{Z_{TEER}\cdot Z_{cell}}{Z_{TEER}+Z_{cell}}$$

This minimal structure reflects the physical topology of the measurement system and shows how paracellular resistance is electrically coupled with capacitive and series contributions. Within this framework, *TEER* cannot be measured directly but calculated. *TEER* is extracted from the system-level impedance and its interpretability depends on how $Z_{electrode}$ and $Z_{background}$ are managed experimentally and analytically [14,19,23].

Although presented in an idealized form (Equation (8)), electrode interfaces rarely behave as ideal capacitors. Surface roughness, heterogeneous double-layer formation, adsorption and microscopic heterogeneity introduce distributed time constants that deviate from purely capacitive behavior. This non-ideal electrode–electrolyte interface response becomes particularly impactful at low frequencies, defined differently depending on the measurement setups and the barrier model, and is commonly approximated by constant phase element (CPE) behavior, which is addressed in Section 3 and Section 4 [17,24].

### 2.4. Electrical Signatures of Cell Barrier

Cell barrier formation is characterized by progressive tight junctions’ organization, leading to an increase in *TEER* and a rise in low-frequency impedance. Over time, as cells proliferate toward confluence, spread, differentiate, and develop specialized membrane structures such as microvilli or cilia, the effective membrane surface area increases. These morphological changes modify the capacitive response of the barrier, affecting both phase behavior and the frequency dependence of impedance (Figure 1B). These capacitive-derived parameters therefore carry biological information related to membrane surface area and morphology, complementing junctional resistance [16,19,25,26].

The frequency-dependent behavior underlying these changes reflects the contribution of cell membrane polarization across the spectrum. Since capacitive impedance decreases with increasing frequency (Equation (7)), low frequencies are dominated by cell membrane capacitance exhibiting high impedance, which forces current to flow primarily through paracellular pathways (Figure 1B). In this region, the signal is largely influenced by resistive contributions associated with *TEER*, while phase remains close to resistive behavior when electrode–electrolyte interface polarization is limited. At intermediate frequencies, reduced capacitive impedance permits partial transcellular coupling, increasing sensitivity to cell membrane surface area and morphology. At high frequencies, capacitive elements become progressively short-circuited and impedance approaches a resistive limit dominated by medium conductivity and volume (Figure 1B) [11,23].

Once cell barrier formation is complete, the resistive component may reach a plateau, reflecting stabilized junctional integrity. Under these conditions, impedance spectra show reproducible low-frequency resistance and a stabilized phase profile consistent with organized tight junctions and cell membrane structure.

Conversely, during cell barrier disruption, *TEER* decreases as junctional integrity is compromised, while cell membrane remodeling and structural changes may alter capacitive contributions. These shifts can modify both magnitude and phase across the spectrum. Impedance evolves as a coupled and dynamic electrochemical signature rather than as a purely resistive variation (Figure 1C), highlighting the importance of spectral interpretation in distinguishing maturation, stability, and destabilization phases.

## 3. Cell Barrier Integrity Measuring Platform Diversity

As established in Section 2, *TEER* is embedded in the complex impedance response of a coupled electrochemical system, described in Equation (8), and the impedance itself is intrinsically frequency-dependent, as demonstrated by Equations (3)–(7). In practice, cell barrier monitoring platforms implement these principles across diverse excitation strategies and electrode configurations, resulting in a wide spectrum of commercially available and research-grade systems. This diversity contributes to the variability of reported *TEER* values in the literature, where the reported quantity does not always correspond to the resistive parameter $Z_{TEER}$ defined in the equivalent representation, shown in Equation (9), but may instead represent the magnitude of the total impedance measured at a fixed frequency.

### 3.1. Mono-Frequency Magnitude-Only Platforms

Table 1 provides a non-exhaustive overview of commercially available cell barrier integrity measuring platforms used for *in vitro* cell barrier characterization and highlights how implementation choices translate into different observables. The first category corresponds to mono-frequency magnitude-only systems. The EVOM and Millicell ERS families commercialized by World Precision Instruments (WPIs) and Merck KGaA are representative of this approach, operating at a fixed frequency, typically around 12.5 Hz and reporting only impedance magnitude. In these early devices, the applied excitation is not a pure sinusoidal waveform but an alternating square-wave signal [10]. Such signals inherently contain multiple frequency components through their Fourier decomposition [27]. However, because these systems do not perform spectral decomposition of the response and do not provide phase information, the measurement remains operationally interpreted as a fixed-frequency magnitude readout rather than as broadband impedance spectroscopy [28]. These instruments have played a historical role in insert-based cell barrier monitoring and remain widely used because they offer a quick and accessible workflow that enables frequent integrity checks during cell barrier formation [9,10]. Their practical value lies in providing an accessible and fast integrity indicator without requiring modeling or dedicated analysis pipelines. Mono-frequency approaches have evolved toward higher-throughput sampling through multiplexed measurement configurations, such as the multi-well *TEER* monitoring platforms developed by Applied BioPhysics for 24- and 96-well formats [29].

**Table 1:** Overview of selected commercially available TEER and impedance spectroscopy (EIS) platforms for *in vitro* biological cell barrier characterization. The table summarizes excitation frequency range, measurable impedance range, phase measurement capability, fitted parameters, and batch measurement capacity (number of samples per acquisition).

Supplier	Model	Frequency Measured (Hz)	Measurement Range (W)	Phase	Fitted Parameters	Sample Measured	References
WPI/Merck KgaA (World Precision Instruments (WPI), LLC—Sarasota, FL, USA)	EVOM2/Millicell ERS-2	12.5	0–10^4^	No	No	1	[30]
WPI (World Precision Instruments (WPI), LLC—Sarasota, FL, USA)	EVOM3	12.5	0–10^5^	No	No	1	[31]
Merck KgaA (Merck KGaA—Darmstadt, Germany)	Millicell ERS 3.0	12.5	0–10^5^	No	No	1	[32]
nanoAnalytics (nanoAnalytics GmbH—Munich, Germany)	cellZscope E	1–10^5^	NA	Yes	*TEER*	6	[33]
	cellZscope+	1–10^5^	NA	Yes	*TEER*, *Ccl*	24	[34]
	cellZscope2	1–10^5^	NA	Yes	*TEER*, *Ccl*	24	[35]
	cellZscope3	1–10^2^	NA	Yes	*TEER*, *Ccl*	Up to 96	[36]
Applied BioPhysics (Applied BioPhysics, Inc.—Troy, NY, USA)	ECIS Z-Theta	10^2^–64.3 × 10^3^	NA	Yes	*TEER*, *Ccl*	8–96	[37]
	*TEER*Z	75	NA	No	No	24 or 96	[29,38]
Locsense (Locsense—Amsterdam, The Netherlands)	Artemis ST	10–10^6^	10–4 × 10^3^	Yes	*TEER*	12–24	[39]
	Artemis MT	10–10^6^	10–4 × 10^3^	Yes	*TEER*	4 × 24 channels (96)	[40]
MIMETAS (MIMETAS — Leiden, The Netherlands)	OrganoTEER	10^−3^–10^6^	NA	Yes	*TEER*	up to 64	[41]
Zurich Instruments (Zurich Instruments—Zurich, Switzerland)	MFIA	10^−3^–5 × 10^6^	1–10^12^	Yes	No	Leader–followers	[42]
	HF2LI	10^−3^–5 × 10^6^	NA	Yes	No	Leader–followers	[43]
Metrohm Autolab (Metrohm Autolab BV—Utrecht, Netherlands)	PGSTAT302N	10^−6^–10^6^	NA	Yes	No	1	[44]

However, the same simplicity defines the metrological limitation of mono-frequency magnitude-only platforms. In all these implementations, the measured quantity corresponds to a reduction of the complex impedance response defined in Equation (3) to its magnitude, in Equation (4), evaluated at a single excitation frequency. Consequently, the reported value integrates paracellular resistance with electrode interface impedance and cell membrane capacitive coupling, whose relative contributions are frequency-dependent and cannot be disentangled without spectral information. This reduction may lead to systematic overestimation or underestimation of the intrinsic resistive contribution associated with paracellular transport, characterized by the *TEER*, depending on the relative balance between resistive and capacitive pathways at the selected frequency. This limitation becomes particularly relevant when cell barrier models are treated as dynamic living electrochemical systems. Cells remodel tight junctions and membranes over time, while the biological and electrochemical environment co-evolves.

Importantly, the deviation between the measured mono-frequency impedance magnitude and the intrinsic *TEER* is not constant but evolves with the system. Changes in cell morphology, confluency, junction organization, and electrode interface conditions may shift the frequency position of the resistive–dominant regime. As a result, a fixed excitation frequency may no longer probe the same physical contributions throughout the experiment, meaning that identical measurements performed at a constant frequency can correspond to different underlying electrochemical states. The limitations of mono-frequency analysis are further explored in Section 4.2.

Under highly controlled and strictly standardized conditions, mono-frequency devices can remain useful for intra-experiment comparisons, but their readout is not inherently reproducible across time, laboratories, or measuring configurations when small variations in electrode placement, cultureware, or interface state are present [18,19,23]. This explains why reported-*TEER* in the literature, using mono-frequency magnitude-only systems, may correspond, in practice, to a configuration-dependent magnitude of the total impedance rather than to the isolated resistive parameter $Z_{TEER}$ defined in Equation (9).

### 3.2. EIS Platforms to Measure Complex Impedance

A second category in Table 1 corresponds to a non-exhaustive commercially available EIS platform, which extends cell barrier monitoring by acquiring impedance over a frequency band and, in many systems, by measuring phases in addition to magnitude. Commercial implementations cover both insert-based and specific culture formats, including OOCs and MPSs, with different compromises between bandwidth, throughput and analytical depth. The nanoAnalytics cellZscope family (cellZscope E, cellZscope+, cellZscope, cellZscope2, cellZscope3) exemplifies automated multi-well EIS approaches in inserts, enabling parallel monitoring while providing *TEER* and, in several models, cell-layer capacitance readouts. Applied BioPhysics ECIS Z-Theta provides broadband complex impedance acquisition in ECIS-format well plates, rooted in impedance-based monitoring of adherent monolayers and extended toward cell barrier interpretation through multifrequency analysis [9,14,26]. Locsense Artemis ST and Artemis MT provide EIS-based monitoring in multi-insert formats, while MIMETAS OrganoTEER targets their own commercial OOC with broadband acquisition and multiplexing, aligned with the need for integrated monitoring in more physiological configurations [11,41]. At the instrumentation end, general-purpose platforms such as Zurich Instruments MFIA/HF2LI and Metrohm Autolab PGSTAT302N provide broad excitation capabilities and high dynamic range, but their use typically shifts complexity toward multiplexing strategy and analysis workflow rather than offering a cell barrier-specific turnkey pipeline [16,17].

Across EIS platforms, the main advantage is not simply the availability of more frequencies, but access to the complex-valued nature of the system response defined in Equation (3). When phase is measured, resistive and reactive contributions can be separated, electrode polarization dispersion can be identified, and frequency regions corresponding to a resistive plateau can be validated before reporting *TEER* as a junction-associated parameter rather than as a magnitude trend [14,23]. This makes EIS approaches particularly relevant for dynamic and physiologically richer configurations, including co-cultures, air–liquid interfaces (ALIs) and perfused microphysiological systems (MPSs), where electrode geometry and electrode–electrolyte interface contributions evolve and make single-frequency magnitude readings intrinsically vulnerable to systematic bias [12,45,46].

Nevertheless, EIS monitoring introduces modeling dependence. *TEER* is not directly measured but extracted from the system-level impedance defined in Equations (3) and (8) and its numerical value becomes conditional on the chosen equivalent representation and fitting constraints. This does not invalidate broadband approaches but requires a structured analytical strategy. Simplified models can be used to support relative integrity tracking and plateau identification, while more detailed models can be designed, experimentally validated and constrained for cases where parameter extraction is required. In this framework, bandwidth selection also becomes part of the metrology as a range around 1 Hz to 100 kHz is frequently appropriate for cell barrier systems, whereas larger culture areas or specific electrode configurations may require extended frequency domains to properly resolve dispersion and background contributions [14,16,17,18].

### 3.3. Electrode Configuration

The device-level diversity summarized in Table 1 must be interpreted together with the non-exhaustive electrode-level diversity presented in Table 2, as excitation strategy alone does not define which fraction of the equivalent structure in Equation (8) dominates the measured response. Electrode material, wiring scheme, and geometry directly modulate the $Z_{electrode}$ and $Z_{TEER}$ are measured. In insert-based systems, STX chopstick-style electrodes typically rely on Silver/Silver Chloride (Ag/AgCl) interfaces and are commonly implemented in a four-wire configuration, historically designed to reduce series contributions, yet still sensitive to placement, surface condition, and electrode–electrolyte interface polarization [10,28,47]. The Endohm concentric electrodes geometry, also based on Ag/AgCl and often used in two-wire mode, improves field symmetry, and reduces positioning variability in inserts, but it does not eliminate electrode polarization effects, which remain embedded in low-frequency magnitude readings unless phase-aware EIS or explicit modeling is implemented. Stainless steel (SlS) concentric electrodes, such as those used in nanoAnalytics cellZscope systems, privilege mechanical durability and cleanability, but their electrode–electrolyte interface polarization deviates from ideal capacitive behavior, motivating non-ideal representations such as CPEs when interpreting broadband data, detailed further in Equation (12) [16,17,24].

**Table 2:** Comparison of the electrodes used for biological cell barrier monitoring in commercially available systems and representative OOCs implementations. The table compares electrode materials, geometry, and compatible cultureware.

Supplier/Developer	Model	Material	Format	Geometry	Cultureware	Key Refs
WPI/Merck KGaA	STX series	Ag/AgCl	4-wire	Vertical chopsticks rods	Culture inserts/commercial OOCs	[10]
WPI	EndOhm series	Ag/AgCl	2-wire	Concentric vertical electrodes	Culture inserts	[10]
nanoAnalytics	cellZscope electrodes	SlS	2-wire	Concentric vertical electrodes	Culture inserts	[33,34,35,36]
Applied BioPhysics	ECIS^®^ Cultureware	Au	2-wire	Bottom-integrated electrodes	ECIS well plates	[48]
	ECIS^®^ *TEER*Z Cartridge	SlS	2-wire	Vertical rods	Culture inserts	[38]
Locsense	Smartlid series	Au	2-wire	Vertical rods	Culture inserts	[39]
MIMETAS	OrganoTEER	SlS	4-wire	Vertical rods	Commercial OOCs	[41]
Douville et al., 2010 [47]	Custom electrodes	Ag/AgCl	2-wire	Horizontal rods	Custom OOC	[47]
Henry et al., 2017 [12]	Custom electrodes	Au	4-wire	Planar electrodes	Custom OOC	[12]
Wei et al., 2023 [49]	Custom electrodes	ITO/Pt	4-wire	Planar electrodes	Custom OOC	[49]
Chebotarev et al., 2024 [46]	Custom electrodes	Au	2-wire	Planar circle in ring and IDEs	Culture inserts	[46]

In well plate-based formats, Applied BioPhysics ECIS integrates gold (Au) electrodes at the bottom surface, providing parametrizable electrode geometry and the possibility to realize various designs, including interdigitated electrodes (IDEs). There is compatibility with automation and throughput, but it also increases sensitivity to surface fouling and to long-term interface drift in media, particularly when monitoring extends over days [12,14,28]. In OOC contexts, electrode integration strategies diversify further. SlS rod-based configurations, including four-wire implementations in commercially available OOCs, facilitate integration and robustness, whereas planar microfabricated electrodes, such as Au planar geometries used in custom OOCs, indium tin oxide (ITO), and platinum (Pt) combinations or circle-in-ring and interdigitated layouts, provide defined current paths and compatibility with microfabrication but expose the measurement to stronger sensitivity to local current density, surface chemistry, and electrode aging [12,45,46,48,49].

The two-wire versus four-wire choice must therefore be interpreted as a design trade-off rather than a universal solution. Four-wire wiring reduces contributions from lead resistance and improves accuracy in low-impedance regimes, but it does not suppress electrode–electrolyte polarization, which remains a dominant low-frequency artifact unless addressed through phase-resolved EIS or constrained modeling. In compact, integrated, and high-throughput systems where the simplicity and robustness of the hardware are prioritized, a two-wire configuration is preferred. Whereas in precision systems for lower geometrical limits, four-wire configuration can be utilized for improved measurement accuracy due to reduced series resistance contributions. The wiring selection is directed by the expected impedance range and the level of analytical resolution required for barrier formation or disruption experiments. Taken together, Table 1 and Table 2 support the central metrological argument of this review. *TEER* is not an absolute quantity directly measured by an instrument, but a configuration-dependent parameter embedded in the complex impedance response of a coupled system, whose interpretability depends on excitation strategy, spectral access, and electrode configuration [14,19,23].

## 4. Discussion of Measurement Limitations and Metrological Bias

Table 1 and Table 2 demonstrate that impedance-based cell barrier monitoring platforms differ not only in their excitation strategies but also in electrode configuration, geometry, and throughput. Although all systems obey the same physical structure defined in Equation (8), their implementation conditions redefine the relative contribution of each term of the equivalent representation and *TEER* values cannot be assumed to be intrinsically comparable across platforms.

### 4.1. Electrodes Geometry and Culture Configuration Dependence of TEER Values

As established in Section 2, *TEER* is embedded in the complex impedance response of a coupled electrochemical system, as defined in Equation (8) and impedance is inherently frequency dependent, as demonstrated using Equations (3)–(7). The measured quantity is therefore not a direct physical constant but a system-level response reflecting the interaction between paracellular resistance, cell membrane capacitance, electrode–electrolyte interfaces, and background contributions.

Any modification of excitation frequency, spectral bandwidth, access to phase information, electrode geometry, or culture configuration alters the relative weighting of $Z_{TEER}$, $Z_{cell}$, $Z_{electrode},$ and $Z_{background}$. In classical cell culture insert systems with approximately perpendicular current fields, normalization to surface area expressed in Ω·cm^2^ partially compensates for culture area scaling. However, this normalization does not correct for non-uniform electric field distribution, lateral current spreading in microfluidic devices, or asymmetric ALI configurations [11,23]. Identical numerical *TEER* values may therefore correspond to distinct physical configurations, distinct current densities, and distinct electrode–electrolyte interface polarization regimes [23,28].

The diversity of platforms summarized in Table 1, together with the electrode configurations detailed in Table 2, illustrates that reported *TEER* values are configuration-dependent outputs of a measurement system rather than intrinsic biological constants. Without explicit reporting of excitation strategy, electrode configuration, and analytical approach, cross-platform comparison remains physically ambiguous [22].

### 4.2. Reduction Bias in Mono-Frequency Magnitude-Only Systems

In mono-frequency magnitude-only systems, impedance is evaluated at a single frequency and reduced to its magnitude according to Equation (4), while the system remains fundamentally complex-valued, defined in Equation (3). Under these conditions, the measured quantity does not isolate the resistive parameter $Z_{TEER}$ defined in Equation (9) but instead reflects the magnitude of the parallel and series combination described in Equation (8).

Since reactive contributions are not separated, cell membrane capacitance and electrode polarization effects remain embedded in the reported value. The assumption that a frequency, such as 12.5 Hz, corresponds to a *TEER*-dominant regime is empirical and cannot be verified without spectral analysis. In simple and stable insert-based configurations, this approximation may provide a useful integrity indicator within a single experiment. However, in dynamic or physiologically complex configurations, including co-cultures, perfused systems or ALI models, the different elements’ contributions likely occur in different frequency ranges, and their magnitude and phase values will change.

Under such conditions, variations in impedance magnitude may arise from capacitive remodeling, electrode surface modification, or environmental fluctuations rather than from true changes in paracellular resistance. The reduction of the complex impedance response to a scalar magnitude therefore introduces a systematic bias that arises not only from the choice of a single observation frequency, but also from the inability of the measurement strategy to access and interpret the full spectral response of the electrochemical system [14,16].

### 4.3. Electrode and Environmental Drift

**Figure 2:**
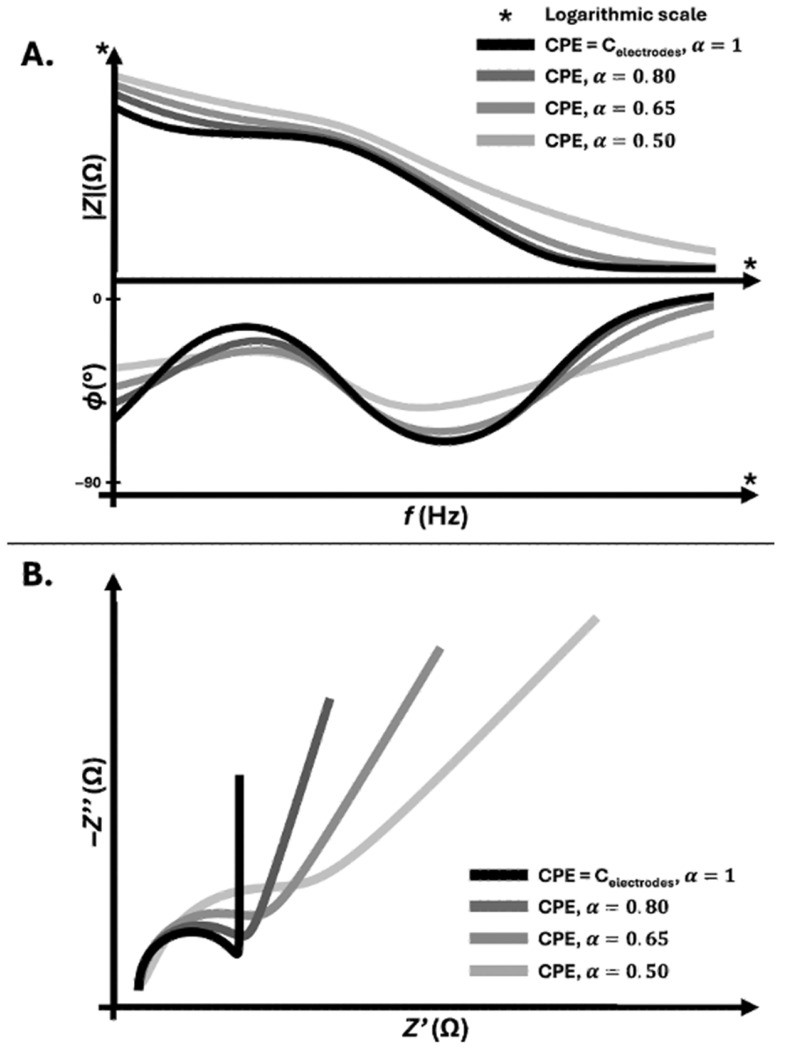
Influence of electrode-interface non-ideality on the impedance response of a cell barrier system. The total impedance was simulated using the minimal equivalent representation presented in Equation (8) where Z_electrodes_ has been modified for a more complete component, Z_CPE_, with identical barrier parameters in all cases. The electrode-interface contribution was modeled as a CPE with varying exponent α, while keeping Q = C_electrodes_. (**A**) Bode representation showing impedance magnitude and phase as a function of frequency. Decreasing α induces phase depression and broadening of the dispersive region. (**B**) Nyquist representation illustrating the transition from ideal capacitive behavior (α = 1) to increasingly non-ideal, distributed responses as α decreases.

Electrode–electrolyte interfaces rarely behave as ideal capacitors described by Equation (7). Surface roughness, adsorption phenomena, distributed reaction kinetics, and ionic diffusion generate a frequency dispersion that deviates from an ideal capacitor. Moreover, these interfaces do not store and release charge uniformly. They exhibit a distribution of time constants associated with heterogeneous surface processes. This behavior results in a phase response that deviates from the ideal −90° of a pure capacitor and cannot be captured by a single capacitance value. This non-ideal electrode–electrolyte interface response is commonly represented by a CPE, whose impedance is defined as:

(12) $$Z_{CPE}\left(\omega \right)=\frac{1}{Q(j\omega {)}^{\alpha }}$$

where Q is a pseudo-capacitance parameter and 0<α≤1 describes the deviation from ideal capacitive behavior. When α=1, the element reduces to an ideal capacitor, whereas when α<1, the phase angle becomes frequency-independent but smaller than 90°, reflecting distributed electrode-electrolyte interface processes [16,17,24]. The impact of electrode-interface non-ideality on the measured response of the full equivalent system defined in Equation (8) was simulated by replacing the ideal electrode capacitance with a CPE of varying exponent α (Figure 2). In all cases, identical values of $R_{background}$, $Z_{TEER}$, and $Z_{cell}$ were used, such that only the electrode-interface behavior was modified to increase its complexity. This shows that decreasing α, corresponding to increasing interface non-ideality, induces a progressive distortion of both impedance magnitude and phase across the frequency spectrum. Phase depression and broadening of the dispersion region are observed, while the Nyquist representation reveals a deviation from ideal capacitive behavior toward distributed, non-ideal responses. Importantly, these variations occur despite unchanged biological barrier properties, demonstrating that electrode-interface effects alone can significantly alter the measured impedance response.

In impedance-based cell barrier monitoring, $Z_{electrode}$ in Equation (8) often exhibits CPE-like behavior, particularly at low frequencies where polarization dominates. In mono-frequency magnitude-only systems, this dispersion is inseparable from the reported impedance magnitude value. Therefore, changes in electrode surface chemistry, protein adsorption, chloride depletion in Ag/AgCl electrodes, or biofouling in microfabricated interfaces may alter the magnitude readout independently of biological cell barrier remodeling.

In EIS devices with access to phase, CPE behavior can be identified through the characteristic phase depression and modeled explicitly. However, introducing a CPE increases model degrees of freedom and requires constrained fitting strategies to avoid parameter correlation with $Z_{TEER}$ or $Z_{cell}$. The presence of CPE-like behavior therefore reinforces the central argument of this review as *TEER* is not directly measured but extracted from a non-ideal, frequency-dependent electrochemical system whose interpretation depends on excitation bandwidth, phase access and electrode configuration.

In MPS and OOC with confined fluidic channels geometries, reduced electrolyte volumes and high surface-to-volume ratios, electrode capacitance dispersion may represent a significant fraction of the total impedance at low frequencies. Without spectral access, the CPE contribution cannot be distinguished from biological resistance changes, further limiting the interpretation of mono-frequency magnitude-only measurements in dynamic living systems.

### 4.4. Modeling Ambiguity in EIS Systems

EIS platforms reduce bias by providing access to both magnitude and phase across frequency. However, *TEER* extraction then depends on the definition of the equivalent representation derived from Equation (8) and on the constraints applied during fitting. Over-parameterized models or insufficiently reported fitting procedures may yield numerically stable yet physically ambiguous solutions. Several sources of uncertainty affect parameter extraction. First, equivalent circuit models are not unique, and different circuit topologies may reproduce similar impedance spectra, especially when the frequency range is limited. Second, strong correlations may exist between parameters, such as CPE elements and resistive components, reducing parameter identifiability and increasing sensitivity to initial fitting conditions. Third, a limited signal-to-noise ratio or insufficient frequency sampling may result in key spectral features required to accurately separate resistive and capacitive contributions. In addition, temporal evolution of the system, including electrode drift and biological remodeling, may alter the impedance response during acquisition, introducing further uncertainty if not properly accounted for in the fitting strategy. As a result, extracted *TEER* values may depend not only on the measurement itself but also on model assumptions, fitting constraints and data quality. This modeling dependency does not invalidate EIS approaches but shifts the metrological challenge from excitation simplification toward model transparency. Explicit reporting of circuit topology, parameter constraints, frequency bandwidth, and validation criteria is necessary to ensure reproducibility and data interpretation. In this context, recent recommendations for minimum reporting standards in *TEER* assays provide an essential step toward harmonization [22].

## 5. Toward an Interpretable and Standardizable Analytical Framework

The diversity of impedance devices, electrode configurations, and acquisition strategies discussed in previous sections and summarized in Table 1 and Table 2, demonstrates that *TEER* values cannot be assumed to be directly comparable across platforms [10]. Differences in excitation frequency, access to phase information, electrode polarization behavior, geometry, and environmental conditions redefine the effective parameters of the equivalent structure introduced in Equation (8). Under these conditions, surface normalization alone does not guarantee physical equivalence between measurements. The objective is therefore to redefine how cell barrier integrity should be extracted and interpreted within three physically coherent and experimentally accessible frameworks, depending on the measurement and acquisition setup.

### 5.1. Relative Integrity Within a Single Measurement Context

A fundamental limitation of mono-frequency magnitude-only measurements arises from the biological nature of *in vitro* cell barrier models themselves. Epithelial and endothelial cell barriers are dynamic living electrochemical systems that continuously remodel junctional organization, cell membrane surface area, cytoskeletal configuration, and ionic transport mechanisms. Cells actively modify their extracellular environment through ion exchange, protein secretion, and extracellular matrix deposition, thereby altering local conductivity and boundary conditions. These biological processes evolve concurrently with electrode interface aging and environmental drift. The measured impedance, $Z_{measured}$, therefore, reflects a temporally coupled system rather than a static circuit. When impedance is reduced to a single-frequency magnitude, resistive and reactive contributions cannot be separated from the electrode–electrolyte interface and environmental evolution. Under such dynamic conditions, mono-frequency magnitude reporting is intrinsically constrained in its ability to isolate junctional resistance during long-term monitoring or in complex OOC platforms [11,50,51].

**Figure 3:**
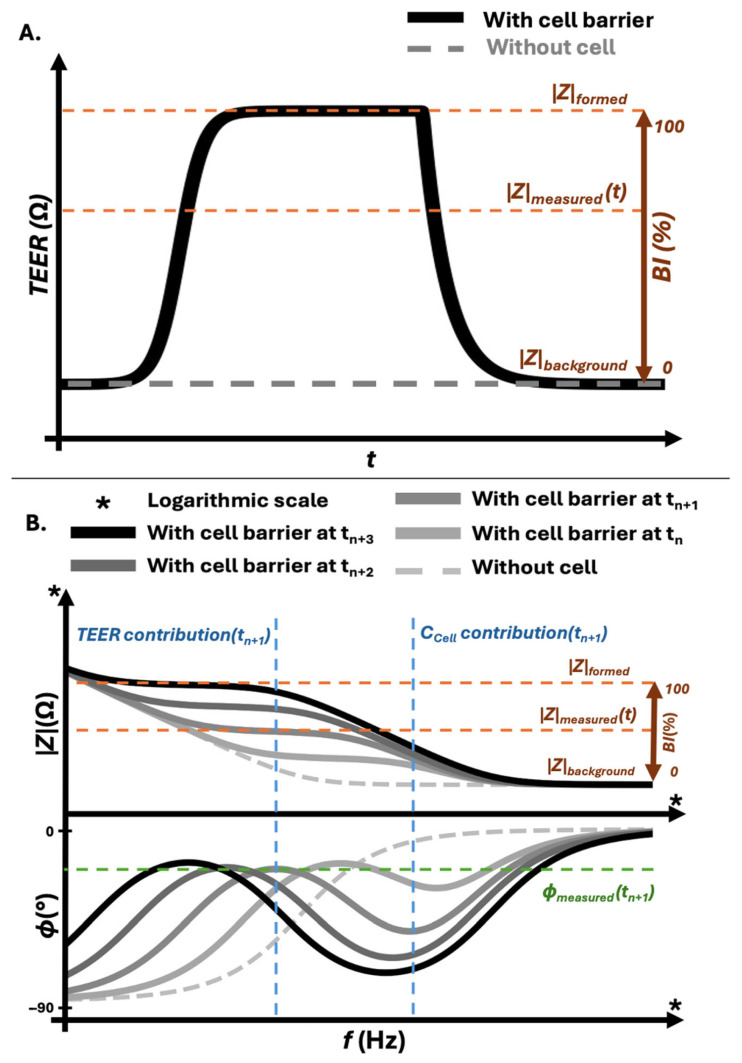
Analytical strategies for impedance-based barrier integrity assessment across time and frequency domains. (**A**) Impedance magnitude over time at a selected frequency. Mono-frequency measurements enable relative barrier integrity tracking by normalizing the measured impedance at a specific time point (${\left|Z\right|}_{measured}\left(t\right)$) to the plateau of the formed barrier (${\left|Z\right|}_{formed}$) and the corresponding background level (${\left|Z\right|}_{background}$). This time-domain approach provides experiment-specific normalization but does not resolve individual resistive and capacitive contributions. (**B**) Broadband impedance spectroscopy represented as a Bode plot. The upper panel shows impedance magnitude as a function of frequency, including the background condition (${\left|Z\right|}_{background}$), the plateau corresponding to the formed barrier ($|{Z|}_{formed}$), and the measured spectrum at a defined time point (${\left|Z\right|}_{measured}(t$)). The high-frequency region provides an estimate of background impedance, while the resistive–dominant plateau reflects barrier formation. The lower panel shows the corresponding phase response over the same frequency range. Combined interpretation of magnitude and phase at the selected time point enables separation of resistive and capacitive contributions within the equivalent circuit and supports model-based extraction of TEER and $C_{Cell}$.

A first analytical level consists of expressing cell barrier integrity as a relative percentage rather than as an absolute *TEER* value (Figure 3A). Using magnitude-only measurements at a given frequency, integrity can be calculated as:

(13) $$Integrity_{\%}=\frac{|Z_{measured}|-|Z_{background}|}{|Z_{formed}|-|Z_{background}|}\times 100$$

In conventional practice, the background resistive contribution is determined from a separate blank sample. To ensure measurement reliability and comparability, the blank must be subjected to the same monitoring protocol and experimental conditions as the cell-containing samples, thereby compensating for electrode aging, medium changes, and environmental drift over time. When normalization instead relies on the tight junctions’ relative resistive plateau reached ($Z_{formed}$), by the same sample and acquisition setup, the electrical environment co-evolves with the cell barrier. This approach partially compensates for slow variations in $Z_{electrode}$ and $Z_{background}$ that would otherwise be misinterpreted as biological change. The resulting metric reflects relative cell barrier integrity within its own measurement context rather than an isolated paracellular resistance. Although this method does not separate the individual contributions of Equation (8), it allows compatible existing mono-frequency devices to estimate cell barrier integrity for single-point measurements and allows the use of previously generated datasets if reporting of measuring and culturing methods enables it.

However, in mono-frequency magnitude-only systems, the measured impedance remains complex-valued, as defined in Equation (3), while only its magnitude is reported, as in Equation (4). Reactive contributions and electrode polarization cannot be distinguished from biological changes. Any capacitive drift due to cell membrane remodeling or electrode aging contaminates the integrity readout and cell barrier integrity monitoring in this specific case becomes frequency-dependent and vulnerable to systematic bias. Therefore, this approach should remain context-specific and be used for short-term measures.

### 5.2. Spectral Validation and Resistive Plateau Identification

A second analytical level integrates EIS magnitude impedance spectroscopy with structured fitting of the equivalent representation defined in Equation (8) (Figure 3B). Through EIS acquisition, *TEER* can be approximated as the resistive component associated with tight junctions’ integrity. More importantly, full-spectrum characterization enables the identification of a resistive plateau region in frequency, where $Z_{electrodes}$ dispersion is reduced. Once this region is validated, targeted mono-frequency monitoring can be performed within the physically justified window in post-experimentation analysis. In this framework, spectral acquisition serves as a calibration step, transforming fixed-frequency monitoring from an empirical convention into a controlled, model-informed choice. This level increases precision while preserving experimental feasibility and it aligns with the operational logic of EIS commercial platforms that extract *TEER* and capacitance through fitting and get a more precise value of cell barrier integrity over long-term monitoring using Equation (13) compared to a fixed mono-frequency measure.

### 5.3. Full Magnitude and Phase from EIS Interpretation

The third analytical level relies on full complex EIS interpretation (Figure 3B). Access to both magnitude and phase allow explicit separation of resistive and reactive contributions and evaluation of the parallel coupling between $Z_{TEER}$ and $Z_{cell}$ described in Equation (8). Phase analysis provides direct insight into whether a given frequency region is dominated by resistive or capacitive behavior, reducing misinterpretation arising from electrode polarization or cell membrane dispersion (Figure 3B). Within this framework, *TEER* is no longer treated as a magnitude reading at an arbitrary frequency but as a model-derived resistive parameter extracted from a validated electrical structure. Since phase information constrains physical interpretation, extracted values become more stable across electrode aging, geometry variation, and environmental drift, supporting a more physically grounded normalization and improved inter-platform comparability.

Using this approach, real-time and long-term monitoring of cell barrier models can be strategically structured. Full-spectrum acquisition performed at defined stages of the experiment enables identification of frequency regions where the impedance response is predominantly resistive. Once this resistive plateau is validated, targeted mono-frequency monitoring can be applied within this optimized window for acute interventions such as drug exposure or mechanical stress. In this configuration, broadband EIS functions not only as an analytical tool but also as a calibration step that informs frequency selection, enabling sensitive detection of cell barrier disruption and recovery while preserving long-term stability of the electrochemical interpretation.

This three-level hierarchy of analysis does not invalidate existing methodologies. Mono-frequency instruments have enabled routine cell barrier monitoring and remain valuable for stable, insert-based applications. Rather, the framework organizes methodologies within a transparent interpretative structure that clarifies the physical meaning and limitations of each analytical level.

In the context of NAMs, this positioning is particularly important because reproducibility and transferability require explicit documentation of measurement conditions. Consistent with emerging minimum information recommendations for *TEER* assays, improved reporting should include excitation frequency or frequency range, phase availability, electrode configuration and materials, geometry and cultureware format, normalization strategy, and the fitting model and constraints when *TEER* is extracted from spectroscopy. By aligning impedance interpretation with both physical principles and biological dynamics, impedance-based cell barrier monitoring can evolve from device-dependent magnitude readings toward reproducible, interpretable, and standardizable integrity assessment compatible with advanced *in vitro* platforms and emerging NAMs-oriented workflows.

### 5.4. Practical Implications for Experimental Design and Data Interpretation

The proposed three-level analytical framework for interpreting impedance measurements into barrier integrity and *TEER* can be applied across different experimental designs involving *in vitro* epithelial or endothelial cell barrier models, including barrier formation monitoring, toxicology assays, drug transport studies, barrier functionality, disease-related alterations, and transport properties through the regulation of ionic fluxes. Typically, cells are cultured on a porous substrate within cell culture inserts or OOC platforms, where electrodes are positioned on either side of the barrier to probe paracellular resistivity. Depending on the instrumentation, measurements may consist of single-frequency magnitude, multi-frequency magnitude, or full complex impedance (magnitude and phase).

From an experimental design perspective, the framework provides a stepwise strategy. Broadband EIS measurements can first be used to identify frequency regions where the impedance response is predominantly resistive, guiding frequency selection for subsequent monitoring, as detailed in Section 5.2. Once this resistive plateau is established, mono-frequency or reduced-frequency acquisition enables routine, real-time tracking of barrier formation, maturation, and response to perturbations. In systems limited to a single frequency where spectral information is not available, plateau identification relies on temporal stabilization of the signal, and interpretation remains relative and context-dependent, as explained in Section 5.1. While using such single frequency systems, researchers must consider electrode and environmental drift effects on measured impedance at a certain frequency (Section 4.3), which could introduce considerable variability in long-duration experimentation. These systems, however, can be effectively used in short-duration experiments, including barrier disruption assays and drug transport assays.

From a data interpretation perspective, the three analytical levels define the degree of confidence and physical meaning of the measurements. Relative integrity metrics (Section 5.1) are suitable for short-term or device-limited use, while spectral validation (Section 5.2) ensures physically relevant frequency selection. For higher accuracy and inter-platform comparability, full EIS analysis including magnitude and phase (Section 5.3) enables extraction of TEER as a model-derived parameter, reducing bias from electrode polarization and environmental drift in long-duration experimentation.

## 6. Discussion

The growing relevance of impedance-based monitoring lies on its ability to integrate seamlessly into NAMs-based research, particularly in the fields of drug toxicity and safety assessment, as a non-invasive, scalable, and physiology-informed functional readout for epithelial and endothelial cell barriers. Its compatibility with real-time acquisition across diverse culture platforms, including inserts and OOC systems [9,10,11], facilitates its widespread adoption in fixed mono-frequency and magnitude-only configurations. However, the parameter commonly reported as *TEER* measured from impedance magnitude is not a direct measure of tight junction integrity. Instead, it represents a resistive descriptor derived from the frequency-dependent real and imaginary response of a coupled electrochemical system. Distinguishing cell barrier integrity from model-extracted *TEER* is therefore essential for correct interpretation and data comparison across *in vitro* and OOC systems within emerging NAMs frameworks.

From a biological perspective, impedance measurements primarily reflect ionic paracellular conductance defined by tight junction organization and ion selectivity. Impedance-derived cell barrier integrity thus quantifies ionic leakiness rather than global molecular permeability. Solute transport assays capture size and transporter-dependent passage and may diverge from impedance readouts when active transport or transcellular mechanisms dominate [3,52]. *TEER* therefore reflects structural ionic restriction but cannot be considered a comprehensive descriptor of cell barrier physiology. This limitation is particularly relevant in complex co-culture or organoid systems. In such cases, impedance measurements should be correlated with complementary readouts to fully characterize barrier functionality. For drug safety evaluation, the combination of TEER data with solute-specific transport assays and imaging can help distinguish between ionic leakiness due to epithelial/endothelial cell barrier disruption and transporter-dependent molecular permeability, thereby refining the interpretation of toxicological mechanisms.

From a physical standpoint, impedance monitoring is intrinsically multivariable. The measured signal integrates junctional resistance, cell membrane capacitance, medium conductivity, electrode–electrolyte interface behavior, and geometric field distribution. These contributions are frequency dependent and may evolve during long-term culture as cells remodel and electrode surfaces age. When reduced to a single low-frequency magnitude, this multidimensional system collapses into a scalar projection. This projection cannot distinguish biological remodeling from capacitive dispersion or electrode–electrolyte interface drift [7,11,16]. Under such conditions, *TEER* becomes configuration-dependent rather than intrinsically transferable. This explains the variability observed across different devices, electrode designs, and culture platforms.

Surface normalization (in Ω·cm^2^), although historically useful in cell culture inserts, does not ensure equivalence across platforms. Small variations unrelated to surface area, such as electrode placement or culture medium volume, may introduce significant variability. Analytical simplifications often neglect geometric factors of both the culture area and electrode configuration. However, changes in geometry can influence electric field distribution, cell barrier formation, and recording sensitivity, even when the nominal surface area remains constant. In microfluidic and miniaturized systems, current density distribution and electric field lines depend strongly on electrode placement and channel configuration [23,53]. Consequently, two systems with identical cell types and nominal surface areas may yield different apparent *TEER* values solely due to geometric differences and electrode-related capacitive effects. *TEER* should therefore not be considered an intrinsic material constant but rather a configuration-dependent system descriptor.

In this context, careful interpretation of impedance data reported in the literature becomes essential. Differences in experimental setups, electrode configurations, excitation strategies, and analysis methods can significantly influence reported *TEER* or impedance values, even for similar biological models. As a result, comparisons across studies should not rely solely on absolute values but must consider the full experimental and analytical context.

To ensure reproducibility and robust interpretation, key experimental parameters should be systematically reported, including cell type and origin, seeding density, culture duration and differentiation state, timing of medium changes, measurement timepoints, electrode configuration and materials, geometry and culture platform, excitation frequency or frequency range, and data analysis strategy. Such information enables researchers to assess the validity of reported measurements, identify potential sources of variability or bias, and determine whether datasets can be meaningfully compared or reused. When sufficiently documented, previously published datasets may also be reanalyzed to enable cross-comparison of devices, experimental setups, and biological models, thereby improving interpretation consistency and supporting the development of more standardized analytical frameworks across platforms.

Phase-resolved EIS provides a more physically grounded framework. Simultaneous analysis of magnitude and phase enables identification of resistive-dominant frequency regions, separation of cell membrane polarization from junctional resistance, and detection of electrode dispersion or aging effects [14,16]. Spectral validation transforms impedance from a single-number readout into a structured diagnostic tool. Nevertheless, EIS does not eliminate ambiguity. Equivalent circuit models remain descriptive representations constrained by measurement bandwidth, signal-to-noise ratio and parameter identifiability. Multiple circuit topologies may reproduce similar spectra when frequency coverage is insufficient or when model constraints are poorly defined.

Under optimized acquisition conditions and adequate spectral coverage, impedance datasets may further support advanced analytical strategies. Machine learning approaches can identify reproducible spectral fingerprints associated with cell barrier maturation, disruption events, or specific culture configurations. These approaches may improve inter-device comparability and compensate for measurement drift. Such data-driven methods may assist in classification, anomaly detection, or early prediction of cell barrier destabilization. However, their reliability depends on robust training datasets, controlled acquisition parameters, and physically interpretable feature selection. Artificial intelligence should therefore augment, rather than replace, phase-resolved impedance analysis.

Electrode–electrolyte interfaces represent a major source of variability in impedance-based cell barrier monitoring. Polarization phenomena, adsorption processes, and surface heterogeneity introduce dispersive behavior commonly represented by CPE [17,24]. Incorporating CPE terms into equivalent circuit models improves fitting and reduces the risk of electrode–electrolyte interface drift misinterpretation to biological disruption. However, CPE inclusion does not confer physical uniqueness; its interpretation depends on transparent reporting of electrode materials, geometry, excitation parameters and environmental conditions. When monitored over time, CPE-related parameters may also provide indirect information on electrode status and stability, potentially supporting the definition of correction factors or criteria for electrode replacement.

Increasing physiological relevance further amplifies system complexity. ALI cultures, dynamic perfusion, breathing mechanics, and co-culture configurations aim to better reproduce in vivo microenvironments [12,13,54]. In many ALI implementations, however, impedance measurement requires transient re-establishment of a liquid electrical pathway, temporarily altering ionic boundary conditions during acquisition. Such measurement-state transitions may influence epithelial physiology or ionic distribution. Impedance must therefore be interpreted within its electrochemical context. Dedicated ALI-compatible electrode integration is essential to preserve culture conditions and maintain the non-invasive nature of impedance monitoring.

Moreover, electrode proximity to the cell barrier can influence both sensitivity and spatial resolution. Distributed electrode networks may enable improved localization of ionic leakiness and enhanced detection of cell barrier disruption during mechanical stress, perfusion changes or shear stress modulation. Such configurations, while technically demanding, may extend impedance analysis beyond global integrity assessment toward spatially resolved functional monitoring.

In co-culture, impedance signals become inherently composite. Extracellular matrices, different cell layers, and three-dimensional *in vitro* models introduce overlapping resistive and capacitive pathways that cannot be uniquely separated without explicit modeling constraints. Impedance remains a bulk measure of cell barrier integrity, providing global ionic conduction information without spatial resolution or layer-specific discrimination. Interpretation in these contexts benefits from correlation with imaging, permeability assays, or other physiological readouts.

These constraints do not limit the value of impedance-based monitoring but rather define its appropriate scope. A fit-for-purpose analytical hierarchy enables its rational use depending on the instrumentation and experimental objectives. Mono-frequency magnitude measurements allow experiment-specific tracking of relative cell barrier integrity and are well-suited for short-term screening or acute perturbation studies. Spectrally informed magnitude analysis improves robustness by identifying resistive-dominant frequency regions, although it remains sensitive to electrode–electrolyte interface drift and capacitive remodeling. In contrast, phase-resolved impedance spectroscopy provides sufficient dimensionality to separate resistive and capacitive contributions over time, supporting long-term monitoring and enabling physically grounded extraction of resistive descriptors. For inter-platform comparison, advanced ALI systems, and future qualification within NAMs frameworks, spectral validation should be considered essential rather than optional. In this context, the proposed framework also provides practical guidance for experimental design and data interpretation, as detailed in Section 5, by linking acquisition strategies to their respective levels of analytical confidence and enabling informed selection of measurement conditions based on the experimental context.

Within this perspective, impedance should primarily be discussed in terms of cell barrier integrity, reflecting ionic permeability across living cellular interfaces. The term *TEER* should be reserved for resistive parameters extracted from validated real and imaginary impedance analysis. As *in vitro* platforms are increasingly positioned as alternatives to animal experimentation, analytical transparency, phase-informed modeling, and harmonized reporting become prerequisites for reproducibility and cross-laboratory comparability.

Future developments may extend beyond conventional equivalent circuit fitting. Machine learning approaches can identify reproducible spectral fingerprints associated with cell barrier maturation or disruption and may assist in compensating for device-dependent transfer characteristics. Integration of phase-resolved features reduces the risk of encoding device bias rather than biological signal. Physics-informed digital twins integrating cultureware, electrode and electrochemical configurations, as well as boundary conditions, may provide predictive normalization across platforms. Such strategies should complement, not substitute, physically constrained impedance analysis. The strength of impedance lies not in delivering a universal constant but in providing a dynamic electrochemical signature of cell barrier integrity that can be integrated with permeability assays, structural imaging, electrophysiology, and label-free optical techniques. In this way, impedance-based monitoring can become not only a core functional readout for *in vitro* platforms integrating epithelial and/or endothelial barriers, but also a bridge between basic *in vitro* biology and regulatory-ready NAMs for drug toxicity and safety assessment.

## Data Availability

No new data were created or analyzed in this study. Data sharing is not applicable to this article.

## Abbreviations

The following abbreviations are used in this manuscript:

NAM	New Approach Methodology
*TEER*	Trans-Epithelial/Endothelial Electrical Resistance
OOC	Organ-On-Chip
EIS	Electrical Impedance Spectroscopy
JAM	Junctional Adhesion Molecule
ZO	Zonula Occludens
CPE	Constant Phase Element
ALI	Air-Liquid Interface
MPS	MicroPhysiological System
Ag/AgCl	Silver/SilverChloride
SlS	Stainless steel
Au	Gold
ITO	Indium Tin Oxide
Pt	Platinum
